# Ultra-Deep Sequencing of Intra-host Rabies Virus Populations during Cross-species Transmission

**DOI:** 10.1371/journal.pntd.0002555

**Published:** 2013-11-21

**Authors:** Monica K. Borucki, Haiyin Chen-Harris, Victoria Lao, Gilda Vanier, Debra A. Wadford, Sharon Messenger, Jonathan E. Allen

**Affiliations:** 1 Lawrence Livermore National Laboratory, Livermore, California, United States of America; 2 California Department of Public Health, Richmond, California, United States of America; The Global Alliance for Rabies Control, United States of America

## Abstract

One of the hurdles to understanding the role of viral quasispecies in RNA virus cross-species transmission (CST) events is the need to analyze a densely sampled outbreak using deep sequencing in order to measure the amount of mutation occurring on a small time scale. In 2009, the California Department of Public Health reported a dramatic increase (350) in the number of gray foxes infected with a rabies virus variant for which striped skunks serve as a reservoir host in Humboldt County. To better understand the evolution of rabies, deep-sequencing was applied to 40 unpassaged rabies virus samples from the Humboldt outbreak. For each sample, approximately 11 kb of the 12 kb genome was amplified and sequenced using the Illumina platform. Average coverage was 17,448 and this allowed characterization of the rabies virus population present in each sample at unprecedented depths. Phylogenetic analysis of the consensus sequence data demonstrated that samples clustered according to date (1995 vs. 2009) and geographic location (northern vs. southern). A single amino acid change in the G protein distinguished a subset of northern foxes from a haplotype present in both foxes and skunks, suggesting this mutation may have played a role in the observed increased transmission among foxes in this region. Deep-sequencing data indicated that many genetic changes associated with the CST event occurred prior to 2009 since several nonsynonymous mutations that were present in the consensus sequences of skunk and fox rabies samples obtained from 20032010 were present at the sub-consensus level (as rare variants in the viral population) in skunk and fox samples from 1995. These results suggest that analysis of rare variants within a viral population may yield clues to ancestral genomes and identify rare variants that have the potential to be selected for if environment conditions change.

## Author Summary

Understanding the role of genetic variants within a viral population is a necessary step toward predicting and treating emerging infectious diseases. The high mutation rate of RNA viruses increases the ability of these viruses to adapt to diverse hosts and cause new human and zoonotic diseases. The genetic diversity of a viral population within a host may allow the virus to adapt to a diverse array of selective pressures and enable cross-species transmission events. In 2009 a large outbreak of rabies in Northern California involved a skunk rabies virus variant that efficiently transmitted within a population of gray foxes, suggesting possible adaptation to a novel host species. To better understand the evolution of rabies virus that enabled this host jump, we applied deep-sequencing analysis to rabies virus samples from the outbreak. Deep-sequencing data indicated that many of the genetic changes associated with host jump occurred prior to 2009, and these mutations were present at very low frequencies in viral populations from samples dating back to 1995. These results suggest deep sequencing is useful for characterization of viral populations, and may provide insight to ancestral genomes and role of rare variants in viral emergence.

## Introduction

Rabies virus (RABV) is one of the most deadly pathogens known and is able to infect a wide variety of mammalian hosts. RABV is present on all continents except for Antarctica and has reservoirs in terrestrial species as well as bats (*Chiroptera*). Although vaccination and antibody therapy is effective in treating known exposures to RABV, an estimated 55,000 human deaths occur annually mostly in developing countries [1]. RABV is a member of the *Lyssavirus* genus, family *Rhabdoviridae*. The genome is composed of negative-sense single-stranded RNA, about 12 kb in size which codes for five proteins- nucleoprotein (N), phosphoprotein (P), matrix protein (M), glycoprotein (G) and polymerase (L). Like other RNA viruses, RABV has a high mutation rate due to the high error rate of the polymerase, thus populations of RABV exist as a mutant swarm, or quasispecies [2]. RABV evolution is believed to be driven predominantly by purifying selection and RABV is not known to recombine [3]–[6].

;Different RABV variants are associated with different reservoir hosts and geographical locations. Typically, interspecies transmission of rabies virus from reservoir to non-reservoir host produces a single fatal spillover event secondary transmission has rarely been observed [7]. For example, a bat variant may infect and cause disease in skunks, but it does not transmit efficiently within the skunk population and skunks would be considered a dead-end host for this variant. The exception to this would be the case of cross-species transmission (CST) where the variant from one species adapts to transmission by a new species [8]. For example, in 2001, bat variant rabies adapted to transmission within the skunk population in Flagstaff Arizona [9], and in 2009 this variant adapted to transmission by foxes [7]. These events demonstrate the capacity of rabies virus for CST which may lead to increased exposure of humans to the pathogen and increase the geographical range of the virus.

Greater than 90 of North American rabies cases occur in wildlife [7], [9], and striped skunks (*Mephitis mephitis*) serve as the most frequent source of terrestrial rabies cases in California [10]. Rabies in striped skunks was first documented in California in 1899 and skunk rabies has been considered enzootic since the 1950s [11]. The Northern Pacific coast region (which includes Humboldt Co.) is unusual in that this is the only region of CA where large numbers of gray foxes (*Urocyon cinereoargenteus*) are known to be infected with the skunk rabies variant [11].

In 2009, the number of rabid foxes in Humboldt County infected with the CA skunk variant increased 356 from an average of 12 per year in the preceding 15 years to 7 in the latter months of 2008 to 38 in 2009 (Annual Reports from California Department of Public Health, Veterinary Public Health Section). In 2009, only 2 skunks were reported rabid in Humboldt County suggesting that rabies infections in foxes had fundamentally shifted from a typical pattern of spillover from skunks to foxes to one resulting from fox-to-fox transmission. The reported numbers underestimate the extent of the outbreak since additional foxes exhibiting unusual or aggressive behavior were euthanized but not tested (S. Chandler, USDA, personal communication). This epizootic of rabies in Northern California raised concerns not only because the primary species involved was gray foxes (*Urocyon cinereoargenteus*) and not striped skunks which are the terrestrial reservoir species in this region, but also because this led to a significant spike in attacks by rabid animals on humans and their pets [12].

The apparent sustained fox-to-fox transmission in this outbreak suggests that CST occurred and enabled this epizootic. We hypothesized that molecular changes in the viral genome would be associated with this event. While phylogenetic data support that rabies viruses have jumped species boundaries historically [5], it is rare and has never been subject to comprehensive genetic analysis at the intra-host population level.

To test our hypothesis and better understand the evolution of rabies, we applied deep-sequencing to 44 unpassaged rabies virus samples from the Humboldt epizootic. Sequence data were generated by two different platforms (Illumina and 454) and by three different commercial services to determine reproducibility. For 40 of the samples, approximately 11 kb of the 12 kb genome was amplified and sequenced using the Illumina platform (the remaining 4 samples were sequenced using the 454 platform only). Average coverage was 17,448 and this allowed characterization of the rabies virus population present in each sample at unprecedented depths.

## Materials and Methods

### Rabies virus tissue samples

The tissue samples used in this study were obtained from the archived collection of California Department of Public Health, Viral and Rickettsial Disease Laboratory (CDPH-VRDL). Gray foxes (*Urocyon cinereoargenteus*) and striped skunks (*Mephitis mephitis*) displaying symptoms of rabies were submitted for rabies testing in the Humboldt Co. Public Health Laboratory between March 2009 and January 2010. Brain tissue samples that were laboratory confirmed to be infected with rabies virus were forwarded to CDPH-VRDL for genetic characterization. Other earlier skunk and fox tissue samples from Humboldt Co. were also available from the CDPH-VRDL archives. As part of routine rabies surveillance in California, the VRDL genotypes rabies-positive samples received from local public health laboratories by RT-PCR and performs sequence analysis on RT-PCR products using forward primer 1066 deg ′′5-GARAGAAGATTCTTCAGRGA-3 and reverse primer 304 targeting a portion of the nucleoprotein (N) gene as described in Trimarchi and Smith (2002) and Velasco-Villa, et al. (2006) [13]–[15]. Approximately 1 gram of brain tissue from foxes and skunks infected with the California skunk rabies virus variant were placed in TRIzol LS Reagent (Invitrogen, Carlsbad, CA) and sent to LLNL for further analysis. RNA was extracted from the tissue sample following the manufacturers protocol.

### Primer design

Approximately 11 kb of the 12 kb rabies virus genome was amplified using degenerate primers (Table S1). Primers were designed to be as sensitive to target strain variants as possible, while still being specific enough to not cross-react with non-targets. Sensitivity was achieved by targeting regions of high sequence similarity, identified through a Multiple Sequence Alignment (MSA) of the target sequences. Specificity was achieved by targeting regions that do not appear to be similar to any other organisms, determined by searching a database of known genome sequences. Primer candidates were selected based on the combined results of the MSA and sequence searches. This technique is a modified version of the approach outlined in Slezak et. al. [16], which accommodates degenerate primer design for diverse target genomes, and places a lower relative priority on primer uniqueness as compared to other known genomes. For rabies virus, which lacks perfect primer-length conservation around the genomic regions of interest, it was necessary to identify degenerate primers for many non-conserved primer regions. From the identified primer candidate regions, which included both perfectly conserved regions and degenerate regions, individual primer pairs were selected which provided overlapping coverage of the DNA being sequenced. Final checks were performed which helped avoid hybridization problems such as primer dimerization.

### RT-PCR, cloning, and sequencing

Reverse transcription was performed using random hexamers and the Superscript III RT reverse transcriptase kit (Invitrogen). The rabies virus cDNA templates were amplified using the Phusion polymerase kit (New England BioLabs, Ipswich, MA), following manufacturers instructions. PCR conditions consisted of 98C for 30 s, followed by 40 cycles of 98C for 15 s, 64C for 20 s, and 72C for 1.2 min. The final cycle was 72C for 10 min.

A plasmid control was generated to determine the error rate of the PCR and sequencing steps as described previously [17]. PCR products were prepared for sequencing using the QIAquick PCR Purification kit (Qiagen, Valencia, CA). Sequencing of an aliquot of a subset of 40 samples was carried out by Eureka Genomics, Hercules, CA using an Illumina Genome Analyzer IIx. Another aliquot of the same samples plus an additional 4 samples were set for 454 sequencing at the Brigham Young University DNA Sequencing Center. Sequencing was performed as described previously [17], [18]. For all samples sequenced by Illumina (paired-end read technology), overlapping read pairs (ORPs), generated by combining short fragment libraries with long sequencing reads, was used to reduce sequencing errors and improve rare variant detection accuracy. Quality filtering procedure was also described in [17], [18].

### Read mapping to reference

The open source read mapping software SHRiMP2, which was shown to have high read mapping sensitivity [19] was chosen for the tools ability to map as many reads as possible in the face of individual errors within each read [20]. All rabies reads were initially mapped to GenBank rabies reference sequence GI:260063801. This reference sequence was used as the common coordinate system for comparing samples and identifying coding frames. Based on a later observation that this newly sequenced rabies virus genome could differ by approximately 9 relative to our selected previously sequenced reference fox rabies sequence, we checked to see if observed error rates would increase by introducing random mutations at 9 of the control reference sequence, however, no noticeable increase in error rates were observed, suggesting that read mapping parameters were able to tolerate this rate of divergence.

The Binomial error model defines the expected number of non consensus bases that should occur given the assumed PCR and sequencing error rate for a given number of observed reads, using a preset P-value (set to 0.01 with a Bonferonni correction). Non-consensus base calls were made when the number of reads with the rare variant exceeded the expected count threshold [17]. The sequencing data used in this study including reads and the analysis files used to make all base calls is available at NCBIs archive BioProject # PRJNA216100.

### Consensus agreement between sequencing runs

Data analysis include 44 samples sequenced using 454 across 10,330 genome positions and 40 samples sequenced using Illumina across 10,379 genome positions. The minimum coverage cutoff was 50. After quality filtering, the mean coverage for the sequencing data was 980 for 454 data and 17,448 for Illumina ORP data, the median coverage was 777 for 454 data and 15,758 for Illumina ORP data (Fig. S1). In total, 10,451 positions of the rabies genome were sequenced by either 454 or Illumina, of these, 10330 positions (98.8 of 10,451 loci) were covered by both platforms, though not necessarily for all samples an additional 36 positions (loci 51995206, 5215, 5216, 52185220, 5224, 95429563) were covered only by 454, and 85 positions (loci 183267) were covered only by Illumina, also not necessarily for all samples. The few disagreements in consensus base calls between 454 and Illumina were resolved either by taking the base call with far superior coverage or omitting the base call from data analysis entirely. In most cases the disagreement was due to low coverage of the loci by one platform (just above the 50 cutoff) compared to coverage by the other platform (500). Hence the final consensus sequences of the two data sets contain no disagreement and can be considered accurate with high confidence.

### Rare variant detection

To differentiate rare variants from sequencing errors, methodologies were developed to measure and control for sequencing and PCR errors and described in Fig. S1 legend [17], [18]. Briefly, all mismatched read pairs in the ORPs were identified as sequencing errors and removed from analysis. Erroneous matching read pairs in the plasmid control were used to estimate the overall PCR error rate [17], [18]. Rates of these two types of errors were then combined in a position-dependent bionomial error model to make variant calls.

## Results

**Figure 1:**
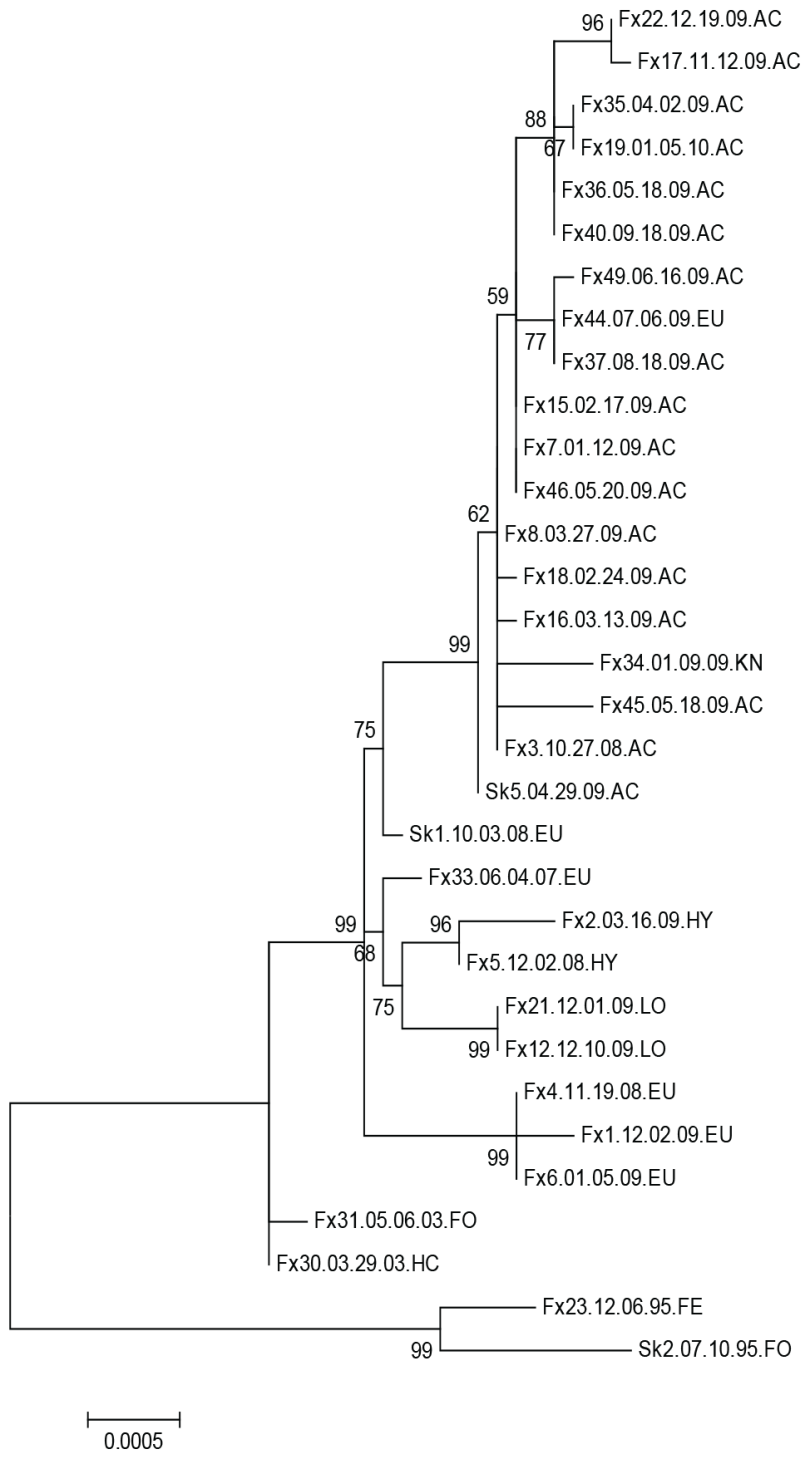
Phylogram generated from genomic sequences of Humboldt Co. skunk variant rabies samples collected between 1995–2010. The evolutionary history was inferred by using the Maximum Likelihood method based on the Tamura-Nei model. The tree with the highest log likelihood (-14363.5660) is shown. The percentage of trees in which the associated taxa clustered together is shown next to the branches. Initial tree(s) for the heuristic search were obtained automatically as follows. When the number of common sites was <100 or less than one fourth of the total number of sites, the maximum parsimony method was used; otherwise BIONJ method with MCL distance matrix was used. The tree is drawn to scale, with branch lengths measured in the number of substitutions per site. The analysis involved 32 nucleotide sequences. All positions containing gaps and missing data were eliminated. There were a total of 9669 positions in the final dataset. Bootstrap values (percentage from 500 replications) are shown for the relevant nodes. Evolutionary analyses were conducted in MEGA5 [28]. Samples in Figures 1, 3 and 4 are labeled according to Sample ID, date and region collected. Regions are abbreviated as follows: AC-Arcata, ER- Elk River, EU- Eureka, FE-Ferndale, FO- Fortuna, HC- Humboldt County (no other geographical information available), HY- Hydesville, KN- Kneeland, LO- Loleta, PP- Patricks Point State Park.

**Figure 2:**
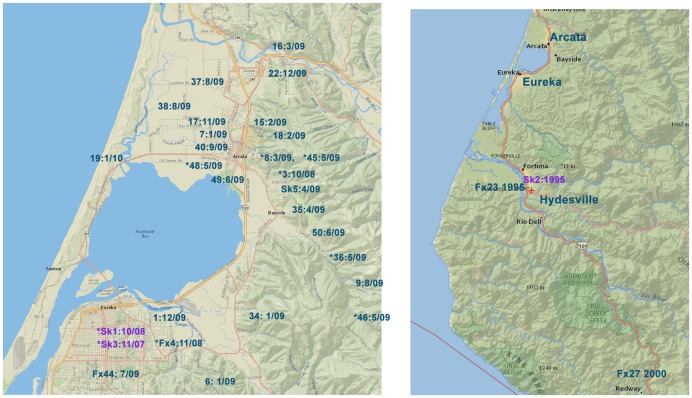
Map of Humboldt Co. showing date and location of rabies samples. Left hand map shows outbreak region, right hand map shows location of pre-outbreak samples. Fox samples are labelled using sample number and collection date. Skunk sample numbers are preceded by Sk. Sample numbers marked with an asterisk indicate exact address of the sample was not available or ambiguous.

**Figure 3:**
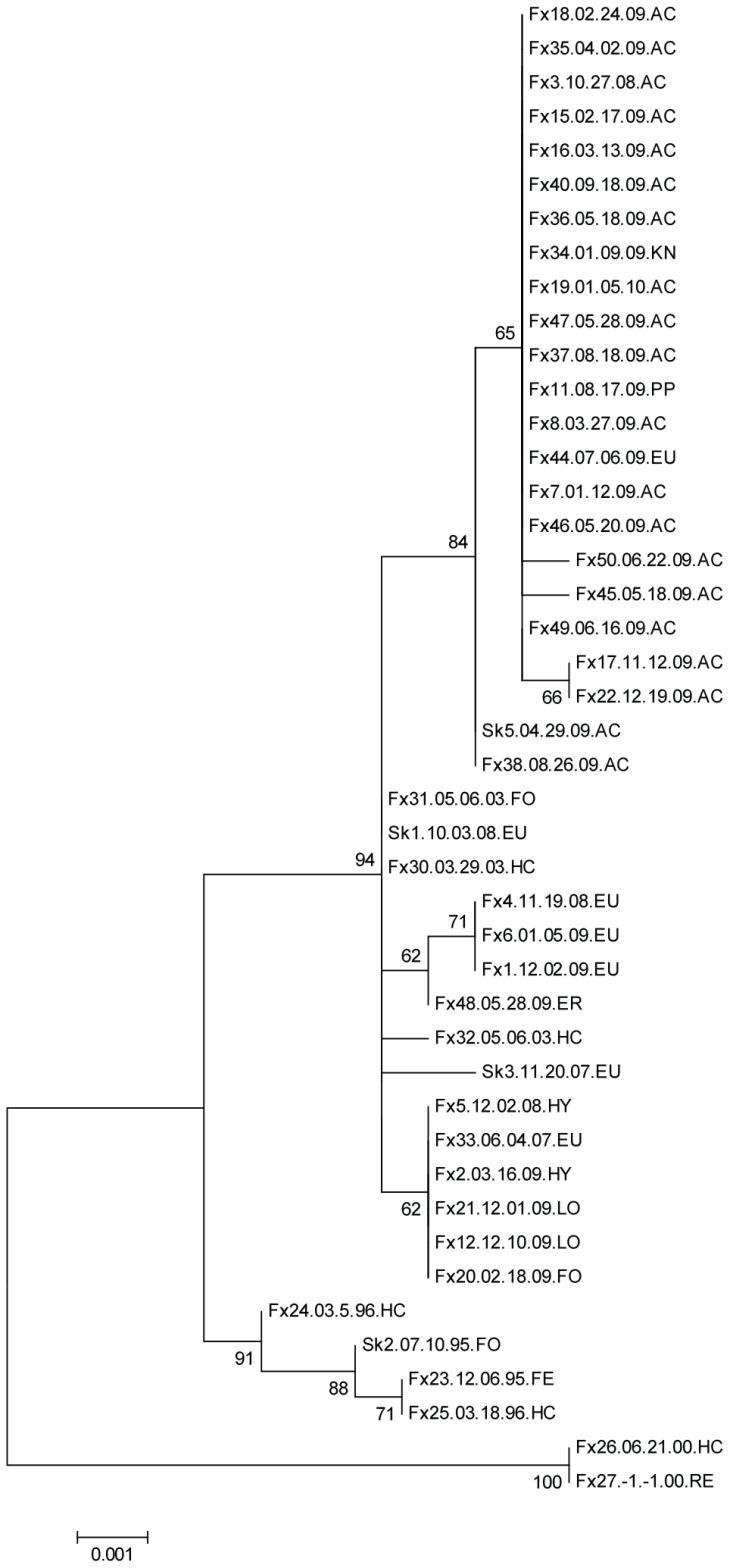
Phylogram generated from G gene nucleotide sequences of Humboldt Co. skunk variant rabies samples. The evolutionary history was inferred by using the Maximum Likelihood method as described for Figure 1. The analysis involved 44 nucleotide sequences. All positions with less than 95% site coverage were eliminated. There were a total of 1498 positions in the final dataset. Samples and regions are labeled as previously described. No data regarding month or day of sample collection was available for sample Fx27, this missing information is shown in the sample label as -1-1.00 to denote the year of collection as being 2000.

**Figure 4:**
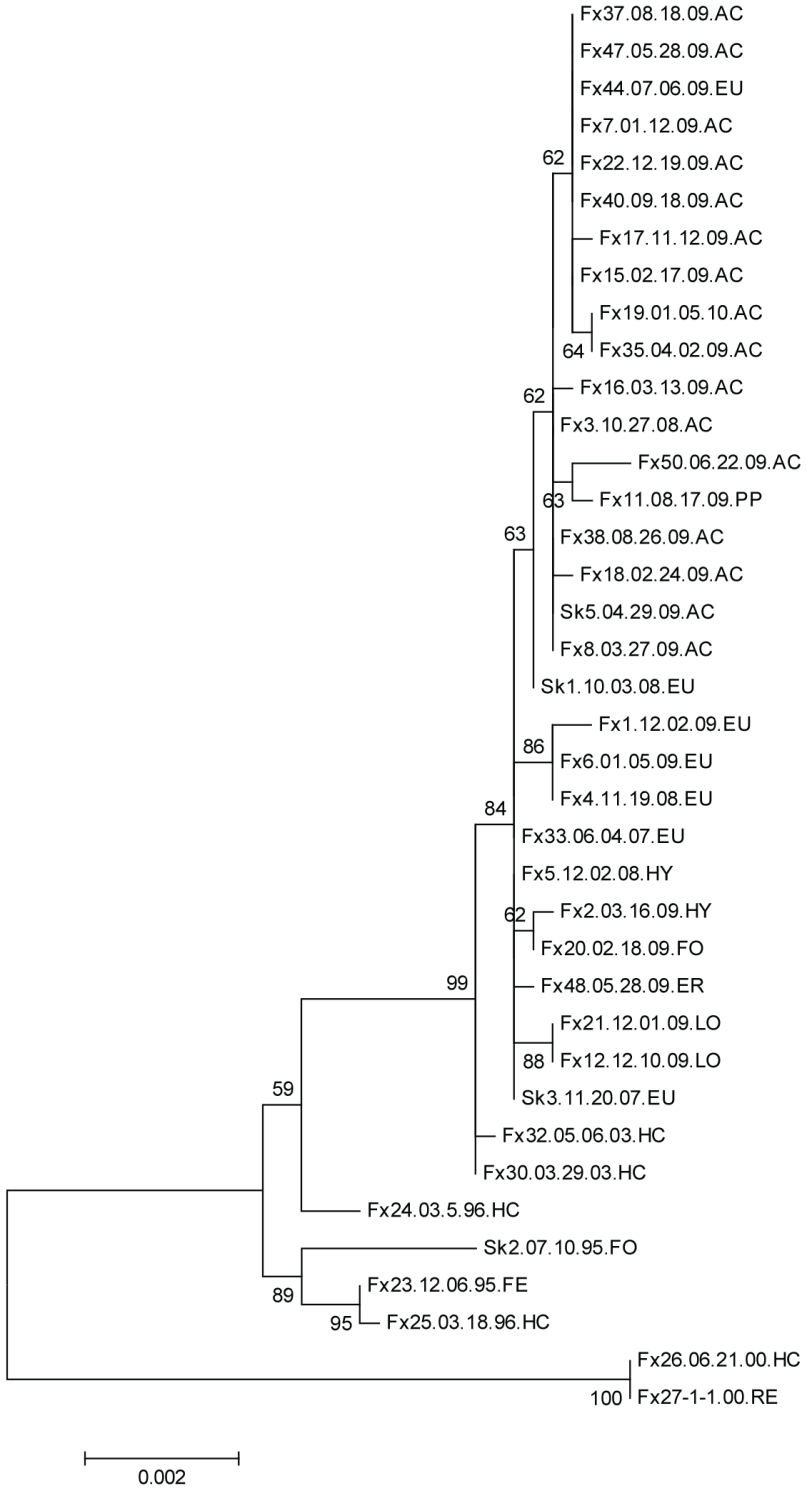
Phylogram generated from L gene nucleotide sequences of Humboldt Co. skunk variant rabies samples. The evolutionary history was inferred by using the Maximum Likelihood method as described for Figure 1. The analysis involved 38 nucleotide sequences. All positions with less than 95% site coverage were eliminated. There were a total of 3979 positions in the final dataset. Samples and regions are labeled as previously described.

Phylogenetic analysis of nucleotide and amino acid data grouped the samples from 2003 with the samples from the 200910 outbreak, thus the 2003 strains are included as outbreak strains (200310) in the subsequent descriptions of results (Figure 1). The samples collected in 2000 grouped separately from all other samples indicating that their haplotype was distinct from that associated with the outbreak, possibly due to geographical separation (Figures 2–4, Table S2).

### Variation across samples in consensus sequence

Among all 10,451 genome positions sequenced, 243 positions contained more than one consensus nucleotide across the samples, and 4 of these positions showed 3 different consensus nucleotides across the samples. These consensus-level variations across the samples occurred in all five genes of the rabies genome as well as four intergenic regions (Fig. S2). The intergenic regions tended have higher rates of consensus-level mutations compared to the 5 genes, with the region between G and L being the most variable (0.06 mutations per nucleotide, Table 1, Fig. S2 B). Separating outbreak samples from earlier samples showed that 67 of these positions remained variable at the consensus level across the samples collected during 2009 and 2010 (Table 1, Fig. S2 C, D). The intergenic region between G and L remained the most variable, followed by the intergenic region between P and M. The M protein had the highest rate of consensus-level variation across all samples, but the G protein had the highest rate of consensus-level variation in the outbreak samples.

**Table 1:** Consensus-level mutations found in 5 coding and 4 non-coding regions of the rabies genome.

Sample timespan	Consensus mutations	N	-	P	-	M	-	G	-	L
**1995–2010**	Per-region	**18**	2	**16**	4	**15**	5	**34**	31	**118**
**1995–2010**	Per-nt	**0.013**	0.022	**0.018**	0.045	**0.025**	0.024	**0.022**	0.060	**0.018**
**2009–2010**	Per-region	**4**	0	**3**	1	**3**	1	**9**	14	**32**
**2009–2010**	Per-nt	**0.003**	0	**0.003**	0.011	**0.005**	0.005	**0.006**	0.027	**0.005**

Across all samples collected between 1995 and 2010, 243 loci were found to contain consensus-level mutations. Across samples collected between 20092010, 57 loci were found to contain consensus-level mutations. Distributions of these mutations are shown in both number per-region and rate per-nucleotide. Dashes in the table heading denote non-coding regions between genes.

### Consensus sequence reconstruction in the genome coding regions

#### N protein

Sequence data was obtained from amino acid 76 through the end of the N protein coding region. One amino acid substitution, F80L, differentiated the 200310 samples from the 199596 samples (Table 2). The samples from 2000 also had an F at residue 80 but differed from the other Humboldt samples at residue 106 with D replaced by G. A subset of the 200910 samples from southernmost region of the outbreak area and collected early in the outbreak (foxes 5, 2, and 20) had an N to S substitution at site 119.

**Table 2:** Amino acid changes according to genomic position, sample collection date, and location.

Gene	Amino Acid No.	Genome Nucleotide no.	1995	1995	1996	2003	2007	20073	2008	2009	2009/10	2009	2000
			Sk2	Fx23	Fx24	Fx31	Fx33	Sk3	Sk1	South Humboldt	North Humboldt	Sk5	Fx27
			Fortuna	Ferndale	na	Fortuna	Eureka	Eureka	Eureka			Arcata	Redway
**N**	**AA 80** 1	**308**	nd	F	F	L	L	L	L	L	L	L	F
**N**	**AA 106**	**387**	D	D	D	D	D	D	D	D	D	D	G
**N**	**AA 119**	**426**	N	N	N	N	N	N	N	N/S^2^	N	N	N
**P**	**AA 30**	**1601**	R	R	R	K	K	K	K	K	K	K	R
**P**	**AA 86**	**1770**	R	R	R	R	R	R	R	R	R	R	K
**P**	**AA 156**	**1980**	E	E	E	E	E	E	E	E	E	E	G
**M**	**AA 21**	**2556**	H	H	H	N	N	N	N	N	N	N	H
**M**	**AA 126**	**2872**	D	D	G	D^3^	D	D	D	D	D	D	D
**M**	**AA 155**	**2960**	I	I	I	I	I	I	I	I	M	M	I
**G**	**AA 12**	**3349**	L	L	L	Q	Q	Q	Q	Q	Q	Q	L
**G**	**AA 427**	**4594**	D	D	D	G	G	G	G	G	G	G	D
**G**	**AA 428**	**4596**	G	G	G	G	G	G	G	G	S	G	G
**G**	**AA 433**	**4612/3**	E	E	E	E	E	G	E	E	E	E	D
**G**	**AA 485**	**4768**	P	P	P	L	L	L	L	L	L	L	L
**G**	**AA 491**	**4786**	R	R	R	R	R	R	R	R	R	R	H
**G**	**AA 500**	**4812/3**	M	T	M	M	M	M	M	M	M	M	V
**G**	**AA 501**	**4815**	S	S	S	P	P	P	P	P	P	P	S
**L**	**AA 17**	**5458**	S	S	S	S	S	S	S	S	S	S	P
**L**	**AA 69**	**5614**	Y	H	H	H	H	H	H	H	H	H	H
**L**	**AA 107**	**5730**	H	H	H	H	H	H	H	H	H	H	Q
**L**	**AA 233**	**6107**	N	N	N	N	N	N	N	N	N	N	S
**L**	**AA 1478**	**9841**	G	G	nd	G	G	nd	G	G	S	S	G
**L**	**AA 1794**	**10789**	I	I	I	I	I	I	I	I	I	I	V
**L**	**AA 1804**	**10820**	V	V	V	V	V	V	V	V	V	V	A
**L**	**AA 1836**	**10915**	V	V	V	V	V	V	V	V	V	V	I

Outbreak samples from South Humboldt County include those from Hydesville, Fortuna, Loleta, and Eureka. Samples from North Humboldt County include those from Arcata, Trinidad, and Patricks Point State Park.

1 No sequence data was obtained for the first 76 amino acids of the N gene.

2 Hydesville and Fortuna samples have S residue at this site.

3 Fx 32 has an E residue at this site.

#### P protein

Phylogenetic analysis grouped samples primarily according to date, with limited grouping according to geography. Only one amino acid change, R30K, differentiated the 200310 samples from 199596 samples (Figure S3). This residue lies in a conserved region of the protein [21], [22]. The samples from 2000 differed from the 199596 at two sites, R86K and E156G, and from the outbreak samples at amino acid 30 with an R at this site rather than a K. Sequence data for the P protein was available for other CA skunk samples in GenBank including one from neighboring Trinity Co. collected in 1997 (V650 CASK) all had an R at site 30, thus the R30K change is unique to the 2003/10 outbreak.

#### M protein

Phylogenetic analysis grouped samples according to collection date and geography. The samples from 1995 and 2000 shared the same amino acid sequence and differed from the 200310 samples with an H to N substitution at residue 21 (Figure S4). The 200910 samples from the northern region (Arcata and Trinidad), including Sk 5 had unique substitution, I155M, as compared to the 199596, 2000, and southern outbreak samples (Table 2).

#### G protein

′′Relative to other regions of the genome (with the exception of 3 and 5 ends), the G open reading frame had low coverage due to difficulty obtaining PCR products for this region (Figure S1). The primers for this project were designed prior to availability of genomic sequence for the CA skunk variant, thus making primer design especially problematic for variable regions of the genome. This likely impacts the number of subconsensus variants detected in this region.

Phylogenetic analysis grouped samples according to date and geography (Figure 3). The sequence data obtained from samples collected between 19951996 differed from the 200310 samples by four amino acid changes in the G protein L12Q, D427G, P485L, and S501P, and each involved a change in polarity (Table 2). One amino acid change, G428S, characterized samples obtained from the southern region versus from the northern region. Samples from Arcata were also defined by nucleotide changes and samples from the southern region of Humboldt Co., Loleta, Fortuna, and Hydesville, grouped together as did most of the Eureka samples (Figure 3, Figure S5). The exceptions were Fx44 from mid Eureka and Fx34 from just east of Eureka, which consistently grouped with the Arcata samples (Figures 1 and 2). The samples collected in year 2000 grouped together and distant from all other samples indicating that these haplotypes were not represented in the outbreak and likely represent spillover events.

#### L protein

Surprisingly, phylogenetic analysis using 3979 nt positions showed the most resolution with subgroups present within both the south and north clusters. Although the samples from 2000 differed from the 199596 and 20032010 samples by six amino acids, the only change observed between the 199596 samples and 20032010 samples was a G1478S in the North Humboldt samples including Skunk 5 (Figure 4, Figure S6). Skunk 2 from 1995 differed from all other samples at site 69 where a Y was present rather than an H (Table 2).

### Consensus sequence reconstruction in the genome noncoding regions

Reads from the noncoding regions were concatenated and consensus data from 454 and Illumina sequencing were compared (Fig. S7). Most samples clustered according to date and location. Noncoding nucleotide data from representative samples from 1995, 2000, 2003, 2007, and 2010 were Blasted to identify similarity to samples in GenBank. Data from all samples had the best match to CA982 (CA skunk from 1994, accession no. JQ685894) with all samples 97 similar except for the sample from 2000, which was 98 similar to CA982.

#### Changes in frequency of rare variants in intrahost viral populations

**Figure 5:**
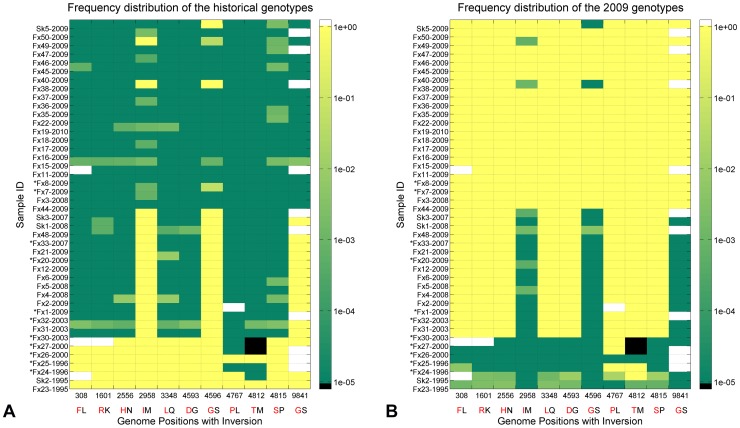
Heatmap showing the frequencies of the historical and outbreak haplotypes at 11 inversion sites for all samples. **A**. Frequency distribution of the historical haplotypes. Abscissa: genome locations where inversion of the consensus and variant amino acids occurred. Letters below the genome locations indicate the two haplotypes: red — consensus haplotypes in the historical samples, black consensus haplotypes in the 2009 outbreak. Colorbar on the right indicates the frequency of the historical haplotypes in log scale. All entries of zero frequency were set to a floor of 10^−5^ so that they correspond to a valid logarithm value of -5 (dark green). Black entries denote samples that contained a third haplotype instead of the two main haplotypes. White entries denote missing samples. Ordinate: sample ID ordered from early to late collection date (though not strictly in the late samples) and south to north collection site. Asterisks indicate samples where only 454 data were available and thus have considerably lower coverage and decreased detection of rare variants. **B**. Frequency distribution of the inversion haplotypes found in the 2009 outbreak. Colorbar on the right indicates the frequency of the 2009 haplotypes in logscale. Dark green entries (1e-5) correspond to haplotype frequency of zero.

Deep-sequencing data indicated that CST likely occurred prior to 2003 since several nonsynonymous mutations that were present in the consensus sequences of skunk and fox rabies samples obtained from 20032010 were present at the sub-consensus level (as rare variants in the viral population) in skunk and fox samples from 1995. For example, both samples from 1995 coded for an Arg at nt position 1601 and a Lys at sub-consensus levels at nt 1601, whereas a fox sample from 2003 coded for a Lys at nt 1601 in the consensus sequence and an Arg at sub-consensus levels, as did the 2009 samples which coded for a Lys at nt 1601 in the consensus sequence (Table 2, Figure 5).

In general, mutations in the consensus sequence could result either from selection for de novo mutations that occurred during host infection, or enrichment of sub-consensus variants that originated from the transmitting host. Since it is difficult to determine de novo mutations without complete sampling of an outbreak, we focused on those consensus mutations that 1) showed inversion between the historical and outbreak samples and 2) led to changes in the amino acids. Historical samples were defined as those collected between 1995 and 2000. Eleven loci were found to have such amino acid inversions, their frequency distributions for the historical and outbreak haplotypes are shown in Figure 5 A and B respectively. Although the frequencies of these two haplotypes sum up to 100 at a given loci in all samples (except for outlier entries colored white indicating no data and black indicating absence of these two haplotypes and presence of a third haplotype), the dominant haplotype tended to be near 100 (values below 100 have a median of 99.86) and the minor variant had extremely low presence (median 0.15, therefore could not be visualized if only one heatmap was presented). The outbreak haplotypes (shown in black letters at the bottom of the heatmaps) began as low frequency variants (0.04 to 6.03) in the historical samples and rose sometime between 2003 and 2009 to become the dominant haplotypes (94100) in most of the outbreak samples (Fig. 5B) while the historical haplotypes (shown in red letters in the heatmaps) went from being dominant (98100 presence) in the historical samples to low level variants (0.051.95) in most of the outbreak samples and even completely disappeared in seven of the outbreak samples (Fig. 5A). These data highlight the dynamic evolution of the rabies genome over time.

Among the 11 inversion loci, 6 were found in G, 2 found M and 1 in each of N, P and L. Two pairs of inversion loci, (4593, 4596) and (4812, 4815) are adjacent amino acids in the G protein. Their frequency distributions in the heatmap suggest that their proximity does not imply linkage. Rather, it appears the historical haplotypes at genome position 4596 (G at AA428) in the G protein may be linked to genome positions 2958 (I at AA155) in M and 9841 (G at AA1478) in L. These 3 loci are too far apart for linkage verification by the short read sequencing method.

Data from Illumina sequencing were used to test the hypothesis that sub-consensus variants that were later enriched to become consensus could be detected at higher frequencies in the historical samples than those sub-consensus variants that did not. Loci shown in Figure 5 where inversion of the dominant and minor variants occurred between the historical and later samples are referred to as inversion sites. The frequencies of sub-consensus variants at the inversion sites were compared to that of variants at non-inversion sites present in the 1995 samples (but not enriched to the consensus level in the 2003/10 samples) to determine if those variants at inversion sites were present in higher numbers in the pre-outbreak samples. The average frequency of sub-consensus variants at inversion sites was slightly but significantly higher than that of sub-consensus variants at non-inversion sites (p0.03, two-sample t-test one-tailed). Samples of individuals from relatively early in the outbreak (prior to July1, 2009) had increased likelihood to have the 1995 consensus haplotype detected at the inversion sites as a sub-consensus mutation, although the difference fell short of statistical significance (p = 0.08).

Some amino acid inversion sites were also associated with other parameters such as geographic location. For example, samples from the south (including Sk 2, and with the exception of Fx 44) had Ile as consensus and either Met (n3) or Thr (n1) as the sub-consensus variant at M protein residue 155 when detected, whereas samples from Arcata (with the exception of Fx 49, 38) had Met as consensus and either Ile (n9) or Thr (n1 Fx 44) when detected. Among the 54 samples (50 foxes and 4 skunks), 14 samples are known to have come from southern regions of the outbreak and 19 samples are known to have come from northern regions. Correlation between the samples geographical locations (categorized simply as north or south) and their consensus nucleotides across the genome showed that mutations at 10 genome positions are highly correlated with geographical origin of the samples (Fishers Exact test, 2-tailed p0.01): 2960, 3872, 4343, 4596, 4748, 5511, 7098, 9405, 9841, 10716. Five of these positions are located in the L-protein, four in the G protein and one in the M protein. Three of these positions, 2960, 4596, and 9841, are loci with inverted mutations between historical and outbreak samples. In particular, 4596 (G428S) is the mutation in G protein that distinguished the northern outbreak samples from the southern ones.

Nonsense mutations generate defective RNAs that may or may not be functional. Stop codon mutations were shown to be maintained within the dengue virus populations, leading to altered viral fitness and thus influencing transmission dynamics [23]. Likewise, it has been shown that human respiratory syncytial virus mutants lacking the G gene are still able to form infectious particles *in vitro* [24]. Multiple mutations resulting in a premature stop codon were observed in the deep sequencing data from this study. Sixty-seven and 116 stop codon mutations were detected across all samples in the 454 and Illumina data respectively, at the mean frequencies of 0.50 and 0.14, respectively. At five genome locations, both 454 and Illumina detected stop codon mutations occurring in the same samples at comparable frequencies. These five positions are 1418 in Fx4 (1), 2790 in Fx23 (1), 2976 in Fx23 (1), 5860 in Fx45 (1), and 8470 in Fx48 (1). These pre-mature stop codons identified by both 454 and Illumina strongly support the presence of defective genomes present in the viral populations of multiple samples. Since many examples of pre-mature stop codons occur with low frequency values it was expected that many Illumina calls would be missed with the lower coverage of 454, hence Illumina results on the stop codon distribution are provided in Figure S8. For example, at locus 7375 almost all samples sequenced by Illumina showed a low frequency (0.10.2) stop codon variant that were not detected by 454.

Across all samples and genome locations sequenced by Illumina, 5146 variants were detected at 2302 genomic locations, or 22 of the 10451 positions sequenced. The frequency of these variants range from extremely rare (0.02 at Fx5 genome location 2130, high detection sensitivity due to high coverage of 196,000, see Figure S1) to very common (38 at Fx 40 genome location 1021, measured at 32 by 454). The mean frequency of the variant pool is 0.3, indicating that the bulk of the variants detected are ultra rare. To examine where in the genome mutations are most likely to occur, only those mutations that occur at 1 in a sample or occur in multiple samples with a cumulative frequency 1 are retained for further analysis. There are 248 loci with such high occurrence/frequency variants (one-percent variants, Figure S9), their distribution across the coding and non-coding regions of the rabies genome (Figure S10) are very similar to that of the 243 mutations occurring at the consensus level (Figure S2). In fact, 58 of these one-percent variants occur at loci with consensus-level mutations.

## Discussion

Although rabies virus has jumped species multiple times in the past [5], [8], [9], [25], the event is relatively rare and deep genome sequence analysis has never been applied to examine the role of intra-host viral populations in such an event. Importantly, the rabies outbreak samples collected by the CDPH were accompanied by epidemiologically important documentation such as exact date and location for most of the samples. Additionally the CDPHs ongoing surveillance efforts provided a unique repository of samples for previous rabies host jumping events, which failed to be efficiently transmitted within the gray fox population. Next generation sequencing technology has recently been used to examine viral heterogeneity of rabies genomes present in infected tissues but has not yet been optimized for detection of rare genotypes (less than 1) [26]. Deep genome sequencing of these recent and past samples allowed us to define the viral mutational dynamics that were associated with a skunk rabies virus variant that efficiently transmitted within a population of gray foxes, suggesting possible adaptation to a novel host species.

Historically, the skunk rabies virus variant present in Humboldt Co. has been detected in foxes more frequently than in any other region of the state but not until 2009 has transmission shifted so disproportionally to the fox population [11]. Our data indicate that the outbreak haplotype responsible is able to be transmitted readily by both skunks and foxes since no genetic changes viral sequence differentiated the skunks and the foxes from the epizootic. These results are similar to those from a study describing the genetic changes associated with the Arizona CST events in that the rabies genotype from the donor species (bats) could not be differentiated from that found in the recipient species (skunks or foxes) [8].

All of the Humboldt samples had an unusual sequence, ETGL, as the final four amino acids at the carboxyl end of G protein. A recent study demonstrated that the last four amino acids in this region impact the virulence of the virus [27]; if the final sequence is ETRL then the virus is attenuated due to induction of neuronal apoptosis. According to this study, virulent, wild type RABV haplotypes have QTRL as the terminal sequence, and do not cause apoptosis of the host cell. The impact of the ETGL haplotype on viral virulence is unclear, although it did not perceptibly impact virulence in skunks and foxes.

No amino acid changes were unique to the 2003 samples as compared to the 2009/10 outbreak samples from the Eureka area and further south (Table 2;) however distinctive nucleotide changes were present in the noncoding and coding regions. Temporal and genetic data indicate that the outbreak began in south Humboldt Co. and spread north to Arcata and three amino acid changes characterized samples from Arcata area and further north (Table 2). Whether or not these genetic changes contributed to the explosive increase in fox rabies that occurred primarily in Arcata during 2009 would require further study (Fig. 2).

It seems likely that a subset of the foxes infected during 2009/10 were part of a fox-to-fox transmission cycle, with limited skunk-to-fox transmission occurring as well. This is supported by the fact that the CDPH collected 24 rabid fox samples from the city of Arcata from October 2008 to January 2010 but only 4 rabid fox samples from Eureka during this time period. During this time there were also 2 samples obtained from rabid skunks, one from Eureka in October 2008 and one from Arcata in April 2009. The collection of 1 skunk to 4 fox samples seen in Eureka is not exceptional there was 1 skunk sample and 4 fox samples collected in Humboldt Co. in 2007 (a nonepizootic year). In 2003, 4 skunk samples were collected along with 10 fox samples. Thus collection of 1 skunk sample and 21 fox samples from Arcata during 2009 is exceptional and one may speculate that this was driven primarily by fox-to-fox transmission. In support of this notion, the online postings of local papers (i.e. Times-Standard, Humboldt Sentinel, Arcata Eye) were searched for articles relating to rabid skunks and foxes during 2009. This search yielded mention of 14 rabid foxes, all were from Arcata.

Although relatively few amino acid changes were associated with the 2003/10 host jump, it is possible that one or more of these changes may have been required for efficient transmission of the virus in the local gray fox population. Despite an extensive search of the rabies virus literature, none of these amino acid changes were described by other studies as being associated with a change in viral phenotype. Transmission studies using reverse genetics are required to identify which genetic changes are responsible for increased transmissibility. Information from this type of analysis may provide important information on the risk of a similar host jump occurring in other regions, including regions where gray foxes overlap with populations of mesocarnivores that have threatened or endangered status.

Both consensus and deep-sequencing data indicate that the haplotype associated with sustained fox-to-fox transmission during the 2009 outbreak occurred prior to 2009 since several nonsynonymous mutations that were present in the consensus sequences of skunk and fox rabies samples obtained from 20032010 were present at the sub-consensus level (as rare variants in the viral population) in skunk and fox samples from 1995 (Figure 5). Analysis of the Illumina ultra-deep sequencing data supported the hypothesis that variants that were later enriched to become consensus could be detected at higher frequencies than variants that did not. In particular, all of the mutations that distinguish the 1995/96 haplotype from the 2003/10 haplotype were present as rare variants in Fx 31, the only 2003 sample for which there is deep sequencing Illumina data available. These results suggest that analysis of rare variants within a viral population may yield clues to ancestral haplotypes and identify rare haplotypes that have the potential to be selected for if environment conditions change.

## Supporting Information

Figure S1 **Coverage of the rabies genome by the two sequencing platforms.** Red: Illumina-Eureka genomics. Blue: 454. Each trace corresponds to coverage for one sample. Color bars at the bottom denote locations of the 5 proteins in the rabies genome: N, P, M, G, L (from the left to right). Illumina data was generated using overlapping read pairs (ORP). ORP analysis is a new method that assesses genome position specific sequencing error. The approach uses paired-end sequencing to sequence a single DNA fragment twice. Sequencing is initiated once from each end of the DNA fragment to produce two distinct sequencer reads. In other applications paired-end sequencing uses larger fragment sizes to ensure that each read generated from the same DNA fragment covers different parts of the molecule to recover more of the original fragment. ORP uses shorter DNA fragments and longer read lengths to maximize the number of bases in the DNA fragment, which are sequenced twice. The redundant sequencing means reads with base calls that disagree with their overlapping pair are recognized as errors and are discarded to effectively lower the sequencing error rate. A position specific base call supported by a read pair can still disagree with the consensus base call leading to detection of rare variants. ORPs provide an important benefit over the alternative of adding higher sequencer coverage since the detected mismatches between read pairs give an empirically derived sequencing error rate, which is specific to each sequencer run.
(DOC)

Figure S2 **Distribution of mutations occurring at the consensus level.** Top: Distribution of 243 loci with variations at the consensus level across all samples. Bottom: Distribution of 67 loci with variations at the consensus level across the 20092010 samples. A. Number of consensus-level mutations at each region in the rabies genome — the N, P, M, G and L genes and the five intergenic regions. B. Rate of consensus-level mutation calculated by normalizing the number of loci in each region by the length of region, yielding per-nucleotide frequency of consensus-level mutations. C. Number of consensus-level mutations found among 20092010 samples. D. Per-nucleotide frequency of consensus-level mutations among 20092010 samples for each genomic region.
(DOC)

Figure S3 **Phylogram constructed from P gene amino acid sequence.** The evolutionary history was inferred by using the Maximum Likelihood method based on the JTT matrix-based model. The tree with the highest log likelihood (-1510.8247) is shown. The percentage of trees in which the associated taxa clustered together is shown next to the branches. Initial tree(s) for the heuristic search were obtained automatically as follows. When the number of common sites was <100 or less than one fourth of the total number of sites, the maximum parsimony method was used; otherwise BIONJ method with MCL distance matrix was used. The tree is drawn to scale, with branch lengths measured in the number of substitutions per site. The analysis involved 44 amino acid sequences. The coding data was translated assuming a Standard genetic code table. All positions containing gaps and missing data were eliminated. There were a total of 262 positions in the final dataset. Evolutionary analyses were conducted in MEGA5.
(DOC)

Figure S4 **Phylogram constructed from M gene amino acid sequence.** The evolutionary history was inferred by using the Maximum Likelihood method based on the JTT matrix-based model. The tree with the highest log likelihood (-631.6601) is shown. The percentage of trees in which the associated taxa clustered together is shown next to the branches. Initial tree(s) for the heuristic search were obtained automatically by applying Neighbor-Join and BioNJ algorithms to a matrix of pairwise distances estimated using a JTT model, and then selecting the topology with superior log likelihood value. The tree is drawn to scale, with branch lengths measured in the number of substitutions per site. The analysis involved 46 amino acid sequences. The coding data was translated assuming a Standard genetic code table. All positions containing gaps and missing data were eliminated. There were a total of 202 positions in the final dataset. Evolutionary analyses were conducted in MEGA5.
(DOC)

Figure S5 **Phylogram constructed from G gene amino acid sequence.** The evolutionary history was inferred by using the Maximum Likelihood method as described for Fig. S3. The tree with the highest log likelihood (-1510.8247) is shown. The analysis involved 44 amino acid sequences. The coding data was translated assuming a Standard genetic code table. All positions containing gaps and missing data were eliminated. There were a total of 490 positions in the final dataset. Evolutionary analyses were conducted in MEGA5.
(DOC)

Figure S6 **Phylogram constructed from L gene amino acid sequence.** The evolutionary history was inferred by using the Maximum Likelihood method as described for Fig. S3. The tree with the highest log likelihood (-3901.1361) is shown. The analysis involved 38 amino acid sequences. The coding data was translated assuming a Standard genetic code table. All positions with less than 95 site coverage were eliminated. There were a total of 1322 positions in the final dataset. Evolutionary analyses were conducted in MEGA5.
(DOC)

Figure S7 **Phylogram generated using nucleotide sequences from the noncoding regions.** The evolutionary history was inferred by using the Maximum Likelihood method as described for Figure 1. The analysis involved 43 nucleotide sequences. All positions with less than 95% site coverage were eliminated. There were a total of 862 positions in the final dataset. Samples and regions are labeled as previously described.
(DOC)

Figure S8 **Distribution of nonsense mutations.** Abscissa: genome positions and gene locations of the stop codon mutations. Ordinate: sample ID. Colorbar indicates frequency of the mutations in logarithm scale.
(DOC)

Figure S9 **Genomic distribution of rabies variants found by Illumina sequencing.** Not all rare variants were included for this graph, only those variants (N = 248) that occurred at above 1% in an individual sample or have a cumulative frequency of >1% over all samples were displayed here. Colorbar on the right indicates the frequency of these rare variants. Variants occurring at above 1% in an individual sample appears as green to red pixels, and variants that occur at lower frequencies in an individual sample but occur in multiple samples such that their cumulative frequencies is >1% appear as blue vertical streaks. Sample IDs are ordered by date. Colored lines on the bottom indicate genomic regions. Positions are not consecutive as only 248 genomic locations are shown.
(DOC)

Figure S10 **Distribution of variants detected by Illumina sequencing across the rabies genome.** Only those variants (N = 248) that occurred at above 1% either in one sample or have a cumulative frequency >1% over all samples are included for this analysis. Dashes at the bottom indicate intergenic regions. A. Number of variants found in each genomic region. B. Per-nucleotide rate of occurrence of these variants in each genomic region.
(DOC)

Table S1 **Primers used to amplify rabies samples.**
(DOC)

Table S2 **Sample metadata.** Sample collection date and location (city or county) are listed. In many cases the street location was obtained for a sample and this information was used for placement of the sample in Figure 2.
(DOC)
