# Supplementary figures and images for: Ultra-Deep Sequencing of Intra-host Rabies Virus Populations during Cross-species Transmission
Source: PLoS Negl Trop Dis. 2013 Nov 21;7(11):e2555. doi: 10.1371/journal.pntd.0002555 (PMC3836733; doi:10.1371/journal.pntd.0002555)

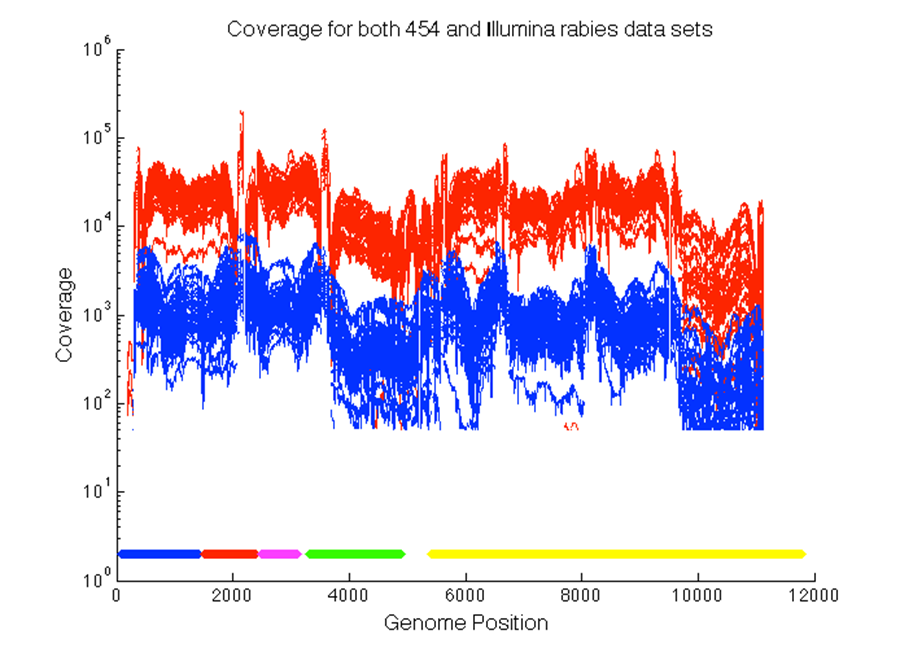


**Figure S1. Coverage of the rabies genome by the two sequencing platforms**.

Supplement: Figure S1 — Coverage of the rabies genome by the two sequencing platforms. Red: Illumina-Eureka genomics. Blue: 454. Each trace corresponds to coverage for one sample. Color bars at the bottom denote locations of the 5 proteins in the rabies genome: N, P, M, G, L (from the left to right). Illumina data was generated using overlapping read pairs (ORP). ORP analysis is a new method that assesses genome position specific sequencing error. The approach uses paired-end sequencing to sequence a single DNA fragment twice. Sequencing is initiated once from each end of the DNA fragment to produce two distinct sequencer reads. In other applications paired-end sequencing uses larger fragment sizes to ensure that each read generated from the same DNA fragment covers different parts of the molecule to recover more of the original fragment. ORP uses shorter DNA fragments and longer read lengths to maximize the number of bases in the DNA fragment, which are sequenced twice. The redundant sequencing means reads with base calls that disagree with their overlapping pair are recognized as errors and are discarded to effectively lower the sequencing error rate. A position specific base call supported by a read pair can still disagree with the consensus base call leading to detection of rare variants. ORPs provide an important benefit over the alternative of adding higher sequencer coverage since the detected mismatches between read pairs give an empirically derived sequencing error rate, which is specific to each sequencer run. (DOC) [file pntd.0002555.s001.doc]

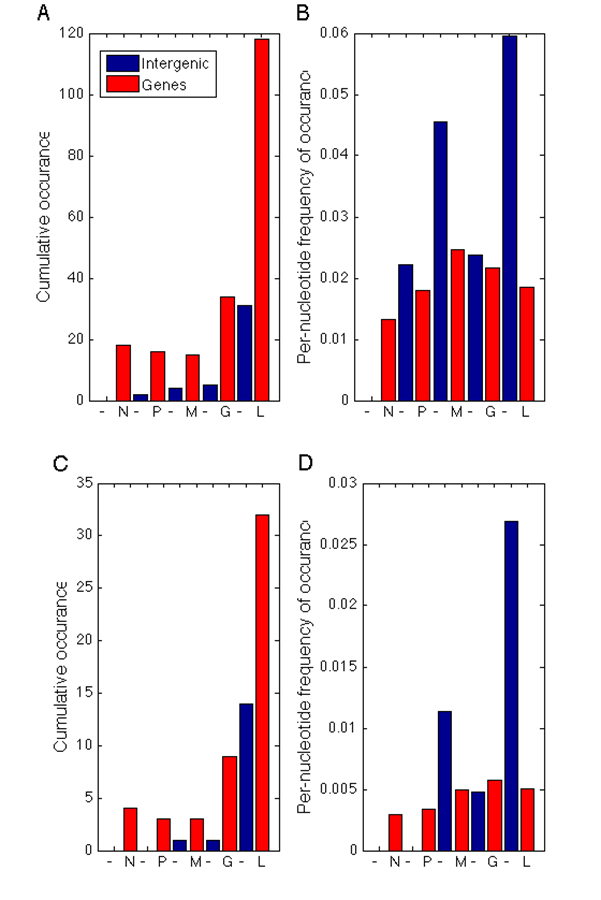


**Figure S2. Distribution of mutations occurring at the consensus level.**

Supplement: Figure S2 — Distribution of mutations occurring at the consensus level. Top: Distribution of 243 loci with variations at the consensus level across all samples. Bottom: Distribution of 67 loci with variations at the consensus level across the 20092010 samples. A. Number of consensus-level mutations at each region in the rabies genome — the N, P, M, G and L genes and the five intergenic regions. B. Rate of consensus-level mutation calculated by normalizing the number of loci in each region by the length of region, yielding per-nucleotide frequency of consensus-level mutations. C. Number of consensus-level mutations found among 20092010 samples. D. Per-nucleotide frequency of consensus-level mutations among 20092010 samples for each genomic region. (DOC) [file pntd.0002555.s002.doc]

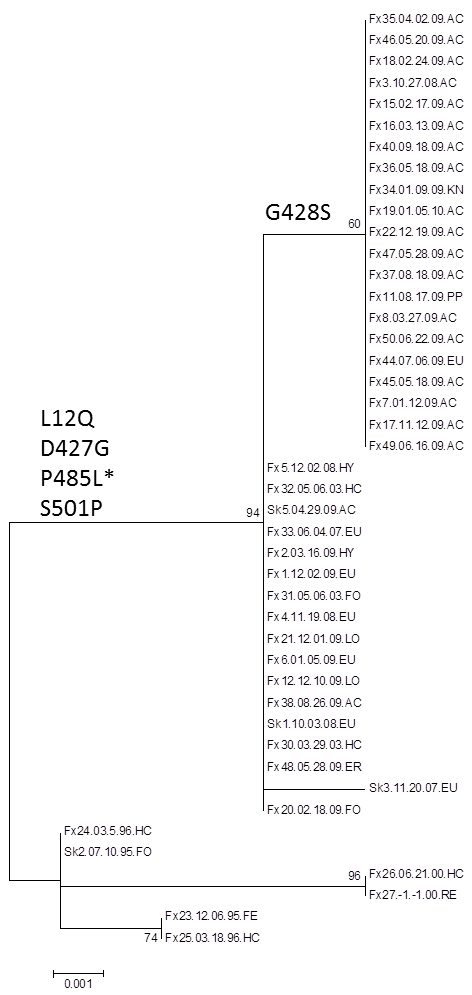


**Figure S5. Phylogram constructed from G gene amino acid sequence**.

Supplement: Figure S5 — Phylogram constructed from G gene amino acid sequence. The evolutionary history was inferred by using the Maximum Likelihood method as described for Fig. S3. The tree with the highest log likelihood (-1510.8247) is shown. The analysis involved 44 amino acid sequences. The coding data was translated assuming a Standard genetic code table. All positions containing gaps and missing data were eliminated. There were a total of 490 positions in the final dataset. Evolutionary analyses were conducted in MEGA5. (DOC) [file pntd.0002555.s005.doc]

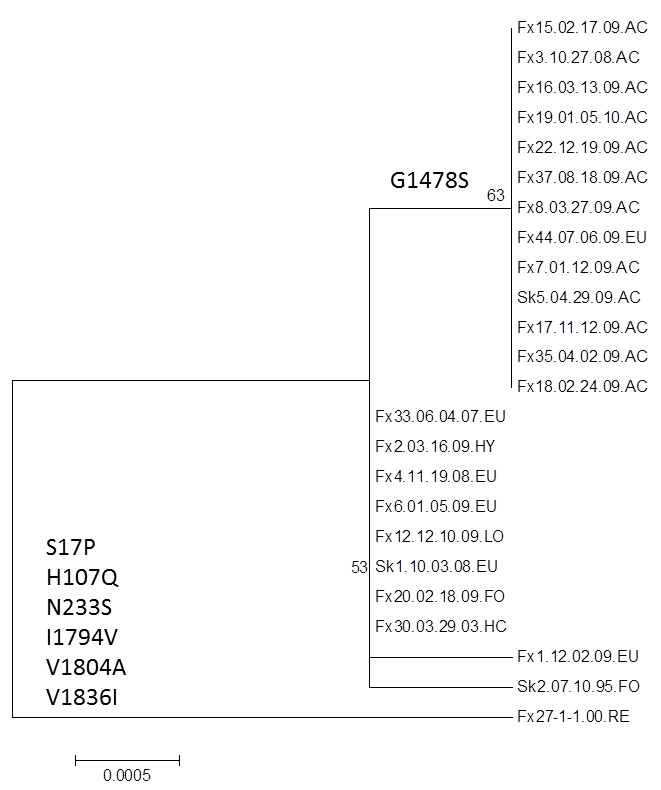


**Figure S6. Phylogram constructed from L gene amino acid sequence**.

Supplement: Figure S6 — Phylogram constructed from L gene amino acid sequence. The evolutionary history was inferred by using the Maximum Likelihood method as described for Fig. S3. The tree with the highest log likelihood (-3901.1361) is shown. The analysis involved 38 amino acid sequences. The coding data was translated assuming a Standard genetic code table. All positions with less than 95 site coverage were eliminated. There were a total of 1322 positions in the final dataset. Evolutionary analyses were conducted in MEGA5. (DOC) [file pntd.0002555.s006.doc]

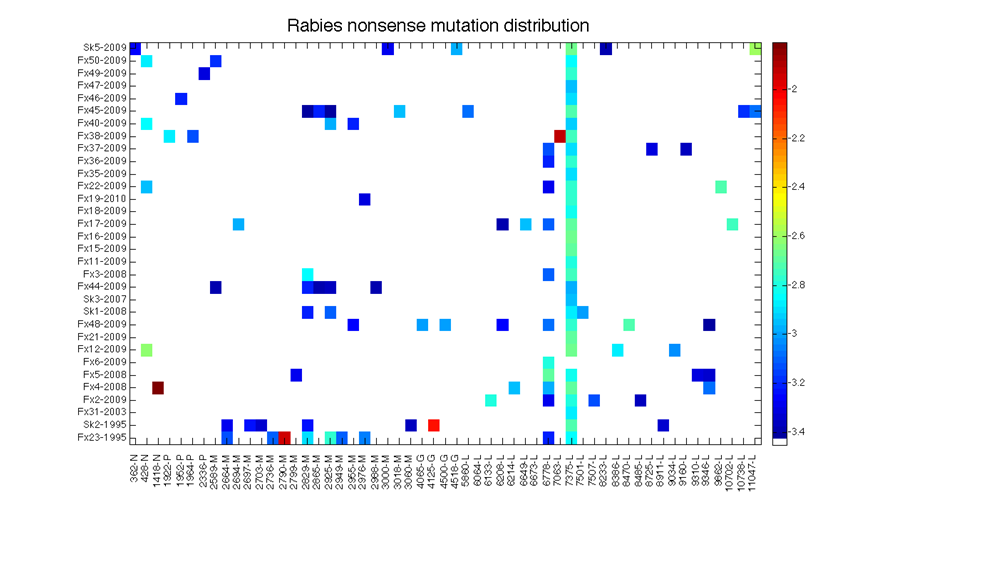


**Figure S8. Distribution of nonsense mutations.**

Supplement: Figure S8 — Distribution of nonsense mutations. Abscissa: genome positions and gene locations of the stop codon mutations. Ordinate: sample ID. Colorbar indicates frequency of the mutations in logarithm scale. (DOC) [file pntd.0002555.s008.doc]

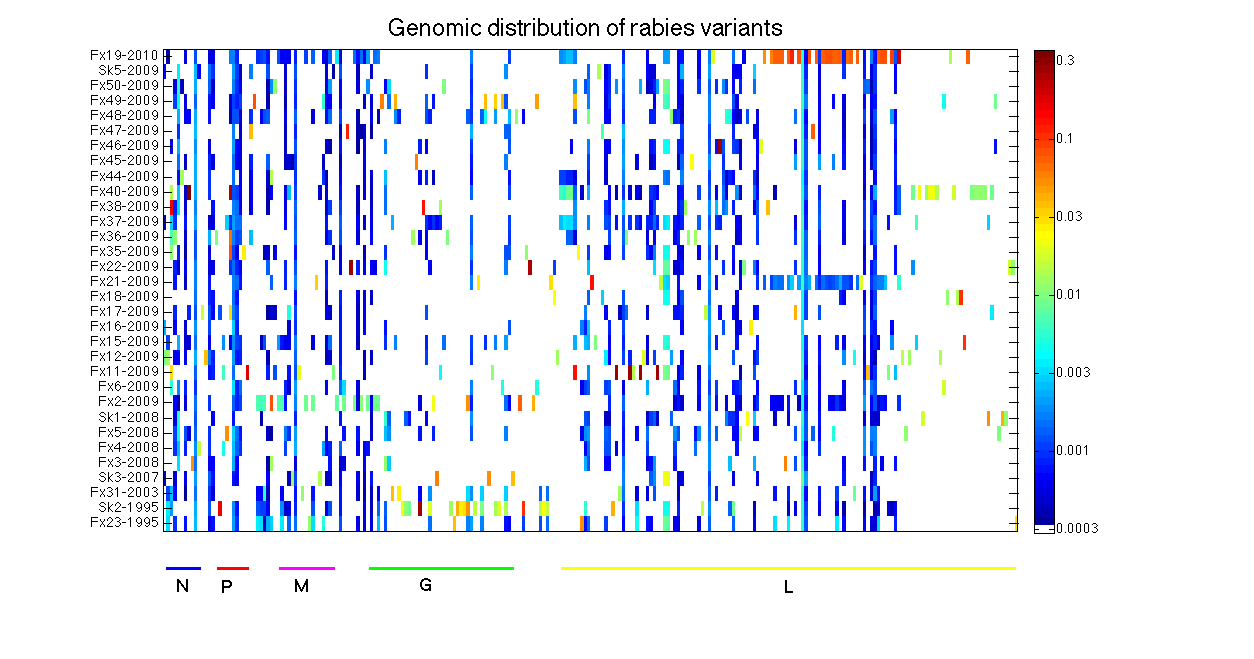


**Figure S9. Genomic distribution of rabies variants found by Illumina sequencing**.

Supplement: Figure S9 — Genomic distribution of rabies variants found by Illumina sequencing. Not all rare variants were included for this graph, only those variants (N = 248) that occurred at above 1% in an individual sample or have a cumulative frequency of >1% over all samples were displayed here. Colorbar on the right indicates the frequency of these rare variants. Variants occurring at above 1% in an individual sample appears as green to red pixels, and variants that occur at lower frequencies in an individual sample but occur in multiple samples such that their cumulative frequencies is >1% appear as blue vertical streaks. Sample IDs are ordered by date. Colored lines on the bottom indicate genomic regions. Positions are not consecutive as only 248 genomic locations are shown. (DOC) [file pntd.0002555.s009.doc]

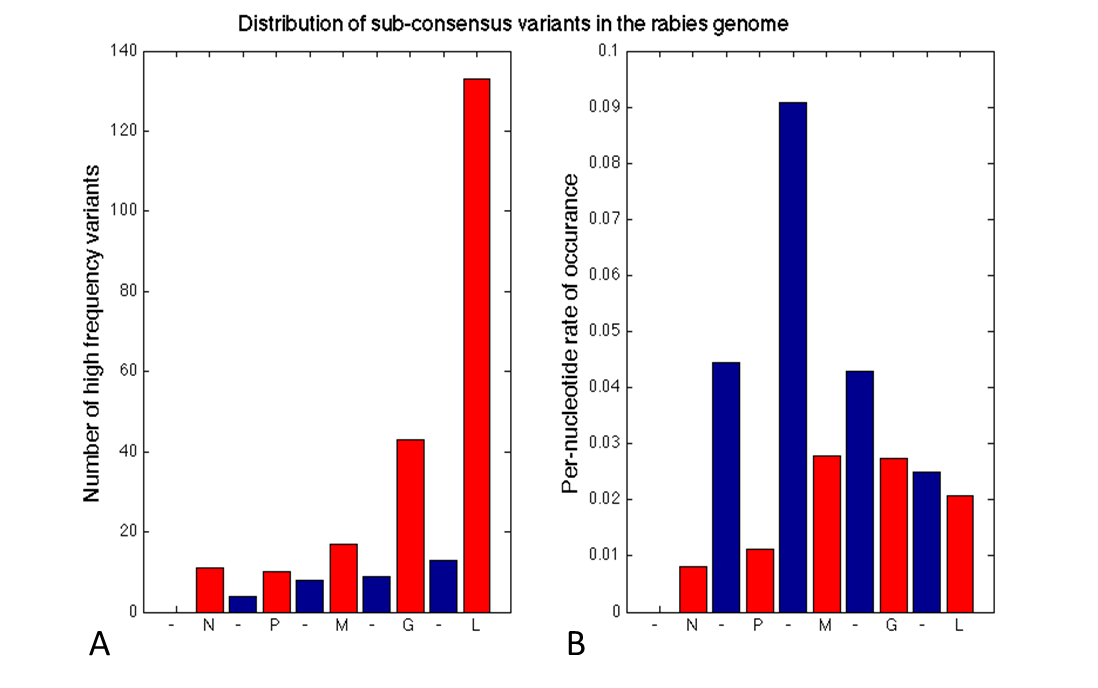


**Figure S10. Distribution of variants detected by Illumina sequencing across the rabies genome.**

Supplement: Figure S10 — Distribution of variants detected by Illumina sequencing across the rabies genome. Only those variants (N = 248) that occurred at above 1% either in one sample or have a cumulative frequency >1% over all samples are included for this analysis. Dashes at the bottom indicate intergenic regions. A. Number of variants found in each genomic region. B. Per-nucleotide rate of occurrence of these variants in each genomic region. (DOC) [file pntd.0002555.s010.doc]
