# Supplementary material for: Ultra-Deep Sequencing of Intra-host Rabies Virus Populations during Cross-species Transmission
Source: PLoS Negl Trop Dis. 2013 Nov 21;7(11):e2555. doi: 10.1371/journal.pntd.0002555 (PMC3836733; doi:10.1371/journal.pntd.0002555)
Supplement: Figure S3 — Phylogram constructed from P gene amino acid sequence. The evolutionary history was inferred by using the Maximum Likelihood method based on the JTT matrix-based model. The tree with the highest log likelihood (-1510.8247) is shown. The percentage of trees in which the associated taxa clustered together is shown next to the branches. Initial tree(s) for the heuristic search were obtained automatically as follows. When the number of common sites was <100 or less than one fourth of the total number of sites, the maximum parsimony method was used; otherwise BIONJ method with MCL distance matrix was used. The tree is drawn to scale, with branch lengths measured in the number of substitutions per site. The analysis involved 44 amino acid sequences. The coding data was translated assuming a Standard genetic code table. All positions containing gaps and missing data were eliminated. There were a total of 262 positions in the final dataset. Evolutionary analyses were conducted in MEGA5. (DOC) [file pntd.0002555.s003.doc]

**Figure S3. Phylogram constructed from P gene amino acid sequence**.
