# Supplementary material for: Ultra-Deep Sequencing of Intra-host Rabies Virus Populations during Cross-species Transmission
Source: PLoS Negl Trop Dis. 2013 Nov 21;7(11):e2555. doi: 10.1371/journal.pntd.0002555 (PMC3836733; doi:10.1371/journal.pntd.0002555)
Supplement: Figure S4 — Phylogram constructed from M gene amino acid sequence. The evolutionary history was inferred by using the Maximum Likelihood method based on the JTT matrix-based model. The tree with the highest log likelihood (-631.6601) is shown. The percentage of trees in which the associated taxa clustered together is shown next to the branches. Initial tree(s) for the heuristic search were obtained automatically by applying Neighbor-Join and BioNJ algorithms to a matrix of pairwise distances estimated using a JTT model, and then selecting the topology with superior log likelihood value. The tree is drawn to scale, with branch lengths measured in the number of substitutions per site. The analysis involved 46 amino acid sequences. The coding data was translated assuming a Standard genetic code table. All positions containing gaps and missing data were eliminated. There were a total of 202 positions in the final dataset. Evolutionary analyses were conducted in MEGA5. (DOC) [file pntd.0002555.s004.doc]

**Figure S4. Phylogram constructed from M gene amino acid sequence.**
