# Supplementary material for: Ultra-Deep Sequencing of Intra-host Rabies Virus Populations during Cross-species Transmission
Source: PLoS Negl Trop Dis. 2013 Nov 21;7(11):e2555. doi: 10.1371/journal.pntd.0002555 (PMC3836733; doi:10.1371/journal.pntd.0002555)
Supplement: Figure S7 — Phylogram generated using nucleotide sequences from the noncoding regions. The evolutionary history was inferred by using the Maximum Likelihood method as described for Figure 1. The analysis involved 43 nucleotide sequences. All positions with less than 95% site coverage were eliminated. There were a total of 862 positions in the final dataset. Samples and regions are labeled as previously described. (DOC) [file pntd.0002555.s007.doc]

**Figure S7. Phylogram generated using nucleotide sequence from the noncoding regions of Humboldt Co. skunk variant rabies samples.**
