# Supplementary material for: Ultra-Deep Sequencing of Intra-host Rabies Virus Populations during Cross-species Transmission
Source: PLoS Negl Trop Dis. 2013 Nov 21;7(11):e2555. doi: 10.1371/journal.pntd.0002555 (PMC3836733; doi:10.1371/journal.pntd.0002555)
Supplement: Table S1 — Primers used to amplify rabies samples. (DOC) [file pntd.0002555.s011.doc]

| Primer Name | Primer Sequence | Start | End |
| --- | --- | --- | --- |
| A_FOR_2_130_RAB | AGACTGGACCAGCTATGGAATCYTG | 331 | 356 |
| A_FOR_3_74_RAB | GATGTTGYTCYTACTTGGCAGC | 275 | 298 |
| A_REV_2_2209_RAB | TCTTGTATAATTTTGAGCAGYTGAAGATGA | 2181 | 2211 |
| A_REV_3_2159_RAB | AGCTTYTCCAAGAAGTACAARTTTCC | 2135 | 2161 |
| B_FOR_1_2056_RAB | CCAGCCCTWGARTGGTCTG | 2057 | 2076 |
| B_FOR_2_2099_RAB | CTGTAGAGGCWGAGATMGCTC | 2100 | 2121 |
| B_REV_1_3622_RAB | TGTCACMACCACRTTCAAAAG | 3602 | 3623 |
| B_REV_2_3687_RAB | TGGAARATGGCWGGCGACC | 3669 | 3688 |
| C_FOR_2_3594_RAB | GTTGGTTATGTCACMACCAC | 3594 | 3614 |
| C_FOR_3_3516_RAB | GGATACATCTCWKCCATAAAAGTGAACGG | 3516 | 3545 |
| C_REV_2_5641_RAB | TCYMGGTCTTACAAAGTTCTGAAA | 5617 | 5641 |
| C_REV_3_5590_RAB | GGBTGARAACAGGAAATAGACCT | 5567 | 5590 |
| D_FOR_1_5420_RAB | CCAGGAGAAGTYTATGATGAYCC | 5419 | 5442 |
| D_FOR_2_5568_RAB | GGTTGARAACAGGRAATAGACCT | 5567 | 5590 |
| D_REV_1_6742_RAB | CCTTATATCAAAACCCAGACATGG | 6718 | 6742 |
| D_REV_2_6757_RAB | AAAACCCAGACATGGCCWCCMAAACATATA | 6727 | 6757 |
| E_FOR_1_6506_RAB | GGTTGTTACAGGCATTGGGG | 6505 | 6525 |
| E_FOR_2_6606_RAB | ACCAGGAGTGTTTAGCRAGYGA | 6605 | 6627 |
| E_REV_1_8291_RAB | GARAGCTTCACWCGCCTTC | 8272 | 8291 |
| E_REV_2_8281_RAB | GGAGAGAGAACACTBGAAAGCTTC | 8257 | 8281 |
| F_FOR_1_8108_RAB | GGKGTGTCTGGAATGTCTCT | 8107 | 8127 |
| F_FOR_2_8036_RAB | ATTYTGAGTGCTGAWGGGGA | 8035 | 8055 |
| F_REV_1_9741_RAB | TRACAAGAATGACAAAYATCAACATCAA | 9713 | 9741 |
| F_REV_2_9660_RAB | GCAARGTCTCCCCKAGAGA | 9641 | 9660 |
| G_FOR_2_9503_RAB | CCAGGTGATTTTGARTCTCTAARTG | 9502 | 9527 |
| G_REV_2_11142_RAB | AGTGAGATGCAGAGRGCYCG | 11122 | 11142 |

**Table S1. Primers used to amplify rabies samples.**
