# Supplementary material for: Ultra-Deep Sequencing of Intra-host Rabies Virus Populations during Cross-species Transmission
Source: PLoS Negl Trop Dis. 2013 Nov 21;7(11):e2555. doi: 10.1371/journal.pntd.0002555 (PMC3836733; doi:10.1371/journal.pntd.0002555)
Supplement: Table S2 — Sample metadata. Sample collection date and location (city or county) are listed. In many cases the street location was obtained for a sample and this information was used for placement of the sample in Figure 2. (DOC) [file pntd.0002555.s012.doc]

| **Sample** | **Date** | **Location** |
| --- | --- | --- |
| **Fx 1** | 12/02/09 | Eureka |
| **Fx 11** | 08/17/09 | Patrick's Point State Park |
| **Fx 12** | 12/10/09 | Loleta |
| **Fx 15** | 02/17/09 | Arcata |
| **Fx 16** | 03/13/09 | Arcata |
| **Fx 17** | 11/12/09 | Arcata |
| **Fx 18** | 02/24/09 | Arcata |
| **Fx 19** | 01/05/10 | Arcata |
| **Fx 2** | 03/16/09 | Hydesville |
| **Fx 20** | 02/18/09 | Fortuna |
| **Fx 21** | 12/01/09 | Loleta |
| **Fx 22** | 12/19/09 | Arcata |
| **Fx 23** | 12/06/95 | Ferndale |
| **Fx 24** | 03/05/96 | Humboldt Co. |
| **Fx 25** | 03/18/96 | Humboldt Co. |
| **Fx 26** | 06/21/00 | Humboldt Co. |
| **Fx 27** | xx/xx/00 | Redway |
| **Fx 30** | 03/29/03 | Humboldt Co. |
| **Fx 3** | 10/27/08 | Arcata |
| **Fx 31** | 05/06/03 | Fortuna |
| **Fx 32** | 05/06/03 | Humboldt Co. |
| **Fx 34** | 01/09/09 | Kneeland |
| **Fx 35** | 04/02/09 | Arcata |
| **Fx 36** | 05/18/09 | Arcata |
| **Fx 37** | 08/18/09 | Arcata |
| **Fx 38** | 08/26/09 | Arcata |
| **Fx 4** | 11/19/08 | Eureka |
| **Fx 40** | 09/18/09 | Arcata |
| **Fx 43** | 08/26/09 | Trinidad |
| **Fx 44** | 07/06/09 | Eureka |
| **Fx 45** | 05/18/09 | Arcata |
| **Fx 46** | 05/20/09 | Arcata |
| **Fx 47** | 05/28/09 | Arcata |
| **Fx 48** | 05/28/09 | Elk River |
| **Fx 49** | 06/16/09 | Arcata |
| **Fx 5** | 12/02/08 | Hydesville |
| **Fx 50** | 06/22/09 | Arcata |
| **Fx 6** | 01/05/09 | Eureka |
| **Fx 7** | 01/12/09 | Arcata |
| **Fx 8** | 03/27/09 | Arcata |
| **Fx 9** | 08/14/09 | Arcata |
| **Sk 1** | 10/03/08 | Eureka |
| **Sk 2** | 07/10/95 | Fortuna |
| **Sk 3** | 11/20/07 | Eureka |
| **Sk5** | 04/29/09 | Arcata |

**Table S2. Sample metadata.**
